# Trends in Hospitalisation for Human Immunodeficiency Virus in a Tertiary Hospital in Dar es Salaam, Tanzania: A Case study

**DOI:** 10.24248/eahrj.v4i1.627

**Published:** 2020-06-26

**Authors:** Grace A Shayo, Tumaini Nagu, Lilian Msele, Patricia Munseri, Columba Mbekenga, Steven Kibusi, Kisali Pallangyo, Ferdinand Mugusi

**Affiliations:** a Muhimbili University of Health and Allied Sciences School of Medicine, Dar es Salaam, United Republic of Tanzania

## Abstract

**Background:**

Reports on systematic evaluation of the impact of antiretroviral therapy(ART) on patients' hospitalisation in Sub Saharan Africa (SSA) and Tanzania in particular are scarce. We aimed at documenting the trends of hospital admissions at Muhimbili National Hospital (MNH) following scale up of free access to ART in Tanzania.

**Methods:**

Records for all admissions at MNH from June 2005 to June 2015 were reviewed. We extracted data from Hospital Information Management System as well as from patients' charts. Data extracted included diagnosis at discharge, reason for admission and thereafter assessed admission trends over the decade. We summarised the data as frequency and percentages. We compared proportions using Chi squared test, P<0.05 was deemed significant.

**Results:**

Overall there were 209,101 admissions during the study period (June 2005 to June 2015) and 7864/209,101 (3.8%) were due to HIV infection. Whereas 598/4,519 (13.2%) of all admissions in 2005 were due to HIV, only 345/13,119 (2.6%) of admissions in 2015 were HIV-related; showing a significant drop over time (*P* value for trend < .001). Generally, females 3887/6679 (58.2%) were more likely to be admitted than males (41.8%). Median CD4 count for admitted HIV patients was 143 cells/µl. Majority of admissions occured in the medical wards 3643/5310 (68.6%). Discharge diagnoses were Tuberculosis 1396/6482 (21.5%), anaemias 1016/6482 (15.6 %), malignancies 789/6482(12.2%), CNS infections 541/6482 (8.3%) and chronic kidney disease 308/6482 (4.8%). Three leading AIDS defining malignancies among hospitalised patients included Kaposi's sarcoma 380/789 (48.2%), carcinoma of the cervix 77/789 (9.8%), and Non-Hodgkin's lymphoma 44/789 (5.6%).

**Conclusion:**

Despite drastic drop of HIV related admissions at Muhimbili National Hospital over the years, the infection remains a problem of the adults, largely females suffering from medical conditions and presenting with severe immunosuppression. Tuberculosis remained the most common opportunistic infection among hospitalized HIV infected patients. Anaemia and cancers became more important causes of admission than was diarrhoea which had been the most common among HIV infected patients in pre- ART era.

## BACKGROUND

At the end of 2018, an estimated total of 75 million people had been infected with Human Immunodeficiency Virus (HIV) and about 32 million people had died of HIV globally since the beginning of the epidemic in 1980. Globally, 37.9 million [32.7 to 44.0 million] people were living with HIV at the end of 2018. The WHO African region remains most severely affected accounting for two-thirds of the people living with HIV worldwide. In Africa, it is estimated that 1 in every 25 adults (3.9%) is living with HIV^1^. Adolescent girls and young women aged 15-24 years have up to eight fold higher rates of HIV infection compared to their male peers and do acquire HIV infection 5-7 years before men^2^.

Diseases related to HIV infections were first reported in Tanzania from Kagera region in 1983^3^. By the end of 1986 cases of acquired immunodeficiency syndrome (AIDS) had been reported from all regions of Tanzania mainland. Parallel to an explosion of hospital admission of patients with full blown AIDS, patients with tuberculosis increased more than three folds. Unprecedented number of patients with other HIV related clinical conditions including Kaposi's sarcoma were also hospitalized in increasing numbers beginning the early 1980's^3^.

Cases of AIDS were first reported at the then Muhimbili Medical Centre (now Muhimbili National Hospital), Dar es Salaam in 1984. Prevalence studies done beginning 1986 among medical admissions, pregnant women, and blood donors in Dar es Salaam showed that HIV infection was wide spread in the general population^4^. In early 1990's the prevalence of HIV among malnourished children was 25% compared to 1.5% in non-mal-nourished children^5^. HIV prevalence among pregnant women in 1984 was 3.2%, reached a peak of 16% in 1989^4^ and then steadily declined thereafter, reported to be 3.3% by 2008^6^.

A high HIV seroprevalence and increased HIV-associated mortality were reported among patients with deep bacterial infections in the medical wards of Muhimbili^7^. A study involving 517 patients hospitalized in the medical wards with fever during February to April 1995 found that 55% of patients tested positive for HIV-1^8^ while 10.5% of surgical admissions in the same hospital from 2001 to 2002 were HIV infected^9^. A total of 118/145 (81%) of surgical patients who confirmed to have blood stream infection were HIV infected^7^. The prevalence of HIV infection among patients hospitalised in the same hospital with pulmonary tuberculosis and/or extra-pulmonary tuberculosis during the 1990s ranged from 33% to 50%^10–12^.

In late 1990s patients who presented with pyomyositis significantly presented with higher rate of HIV seropositivity (62%) than were non-patient controls (12%).^7^

In the 1990s free ART were not provided to a large part of Sub Saharan Africa (SSA) including Tanzania due to high cost of the antiretroviral drugs and lack of adequate laboratory capacities. Beginning 1996 combination therapies with three antiretroviral drugs were found to be more effective in lowering HIV viral load thus became standard of care for patients with HIV disease including AIDS in the industrialised countries.^13,14^ In early 2000s physicians in Tanzania prescribed first line ART drugs in different combinations including dual therapy with zidovudine and lamivudine or triple therapy with Zidovudine, Nevirapine and Didanosine or Zidovudine, Nevirapine and Stavudine. It was not until 2005 that HIV treatment in Tanzania was standardized to 3 drugcombinations after the release of the first HIV guideline. The use of 3 drug combinations resulted into decreased hospitalisation and mortality rates with resultant increased life expectancy in different countries.^15–17^ Moreover, during the ART era, there has been a shift of causes of deaths from AIDS defining to non AIDS defining illnesses.^18,19^

In 2004 Tanzanian government launched a countrywide provision of ART to AIDS patients, commencing with a pilot clinic at Muhimbili National Hospital (MNH). National data for HIV prevalence among adults aged between 15 years and 49years gradually fell from 7% to 5.1% in 2003/4 and 2011/12 respectively.^20,21^ The 2016 National HIV survey found a National prevalence of HIV at 5.0%, a similar figure as that of 2011/12^22^. With increased access to ART the average number of personal admissions due to HIV in various parts of the world fell by 39% early in 2000 and the overall number of hospital admissions due to HIV fell by about 10% to 65%. This study therefore intended to document the trends of hospital admissions at MNH following free access and wide scale up of ART services in Tanzania.

## METHODS

### Study Design, Site and Data Sources

**FIGURE 1.**
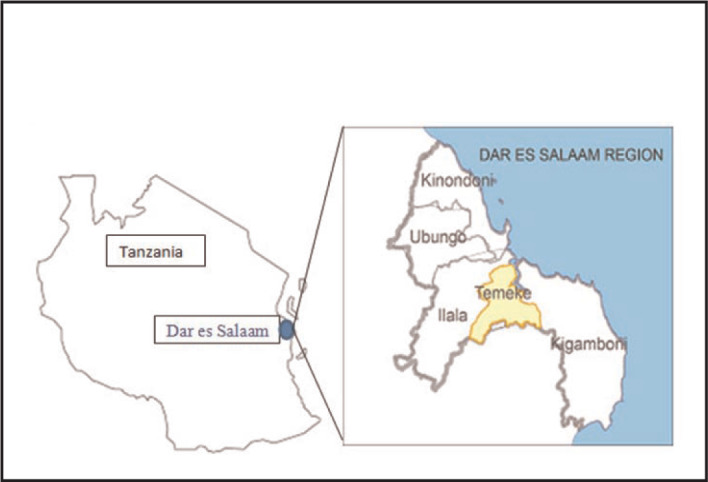
Location of Dar es Salaam City and Its Districts within Tanzania

This hospital based cross sectional descriptive study aimed at describing trends in admission rates and reasons for admission over time following free ART access in November 2004. Hospital based data was collected at Muhimbili National Hospital (MNH), the largest tertiary hospital in the country and a university teaching hospital situated in the city of Dar es Salaam. MNH is a 1,500 bed facility, attending 1,000 to 1,200 outpatients weekly, and admitting 1,000 to 1,200 inpatients per week. It receives referral cases predominantly from within the city of Dar es Salaam and from all over the country. Figure 1 shows the location of Dar es Salaam city and its districts within Tanzania. We used electronic hospital information system and patients' paper files as source of the collected information on hospital admissions and the reasons for admission respectively.

### Data Collection Procedures

Since electronic data was available from June 2005, we collected data on hospital admissions from June 2005 to June 2015. Information on date and number of admissions was extracted from the electronic records. We reviewed patients' paper files to collect information on causes of admissions and discharge diagnoses. Each admission was treated as a separate entity and analysed as such.

For this study, we included everyone who was admitted in the hospital in the specified time frame and we had no exclusion criteria. We obtained file registration numbers of all admitted HIV infected patients and thereafter retrieved the respective files from the records department. The investigators reviewed each of the patients' files. Clinical record forms were used to record the extracted data from hospital records. The information collected included socio-demographic data, HIV status, WHO HIV staging, CD4 cell counts, and status of antiretroviral therapy use, discharge diagnosis, duration of hospital stay and admission outcome (discharge or death). HIV status was ascertained by looking at the case notes. Some patients were already known to be HIV infected at the time of admission while some were tested and diagnosed with HIV infection during an index admission.

### Statistical Analysis

Absolute numbers of admissions due to HIV over the study period are presented graphically to show the general admission trends over time. Proportions of HIV admissions overtime were calculated by dividing the total number of admissions due to HIV per year by the total number of hospital admissions in that particular year. We used STATA 12.0 statistical package (StataCorp, Texas U.S.A) to analyse the data, and Chi Square statistics test (χ^2^) was performed to compare proportion of admissions due to HIV over time. *P*<.05 was considered significant. Causes of admissions are presented as absolute numbers as well as proportions

### Ethical Approval and Consent to Participate

The study obtained ethical clearance from the Muhimbili University of Health and Allied Sciences (MUHAS) Institutional Review Board, reference number MU/DRP/AEC/Vol.XVIII/139. The MNH management authorized unrestricted access to hospital records to extract required information for this study. The clinical request form (CRF) only included hospital registration numbers and patient's initials and not names. CRFs were kept locked in filing cabinets and made accessible to authorized personnel only.

## RESULTS

**FIGURE 2.**
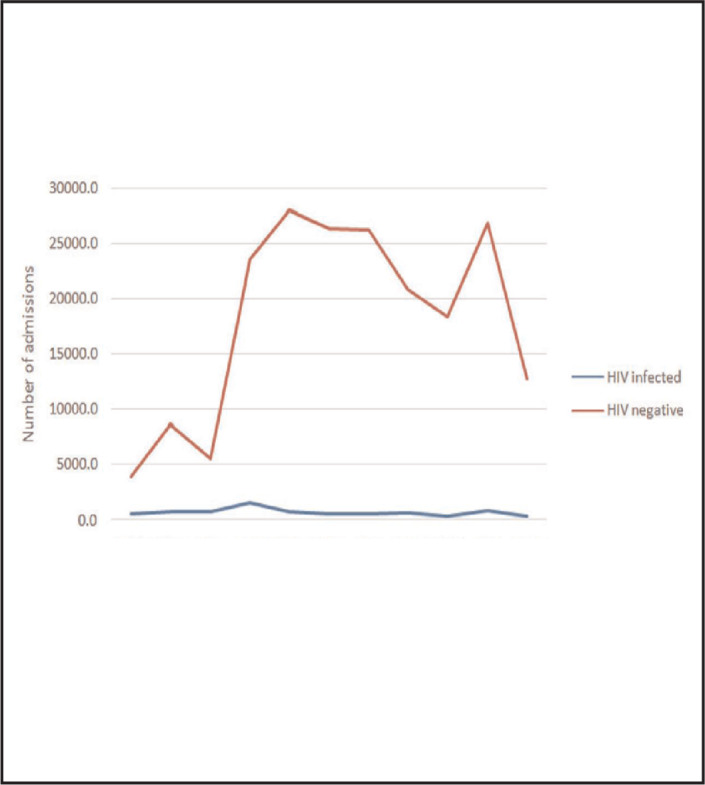
Absolute Number of Admissions from June 2005 to June 2015 Years

In the span of 10 years, there were a total of 7,864 (3.8%) admissions due to HIV out of 209,101 total hospital admissions. Absolute numbers of admissions in the hospital over the years were as shown in Figure 2. Admissions due to HIV dropped drastically from 13.2%(598/4519) in 2005 to 2.6% (345/13,119) in 2015 (See Table 1) [Chi square=1748.473:*P*<.001]

**TABLE 1.** Proportion of HIV-related admissions from June 2005 to June 2015

Year	Total Hospital admissions	HIV-infected	
		Number	Percentage
2005	4,519	598	13.2
2006	9,463	775	8.2
2007	6,238	723	11.6
2008	25,201	1611	6.4
2009	28,734	736	2.6
2010	26,988	587	2.2
2011	26,774	561	2.1
2012	21,578	703	3.3
2013	18,742	369	2.0
2014	27,745	856	3.1
2015	13,119	345	2.6
TOTAL	209,101	7,864	3.8

**FIGURE 3.**
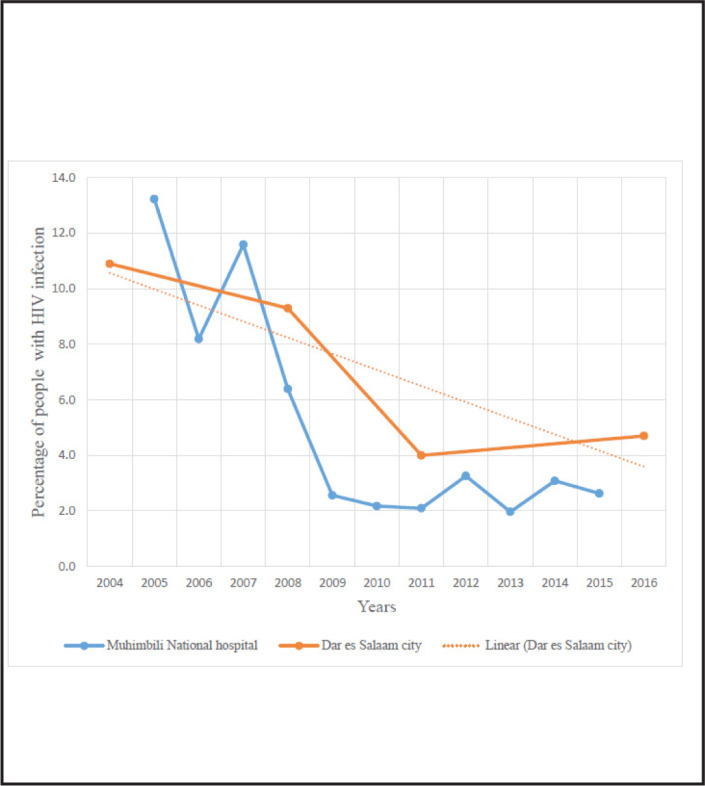
Comparison of Hospital Base (MNH) and General HIV Prevalence in the City of Dar es Salaam

Comparison of hospital base (MNH) and general HIV prevalence in the city of Dar es Salaam is depicted in Figure 3. Decline of HIV prevalence was observed in both hospital and city surveys.

Out of 7864 HIV-related admissions, we managed to find 6874 (87.4%) patients'files, from which data was extracted. Females constituted 3887/6874 (58.2%) of the total HIV related admissions. Of the 6874 admissions only 5310 had recorded ward of admission. Majority of HIV related admissions were commonly found in medical wards3643/5310 (68.6%). Other wards where HIV patients were admitted included surgical 795/5310 (15.0%), paediatric 531/5310(10.0%), gynaecology 290/5310 (5.5%), 19/5310 (0.4%) emergency medicine/ICU, 21/5310 (0.4%) obstetrics and 11/5310 (0.2%) psychiatry. Of the HIV related admissions, majority were on ART (61.6%) and adults (87.6%) aged ≥18 years (See Table 2). Median CD4 cell counts (IQR) was 143 cells/µl(ranging from 48 cells/µl to 320cells/µl) whilst median (IQR) hospital stay was 7 days (ranging from 3 to 14days) (Not shown in the Table).

**TABLE 2.** Characteristics of HIV Infected Patients Hospitalised in Muhimbili National Hospital From June 2005 To June 2015, N=6874

Characteristic		Missing numbers (%)	Frequency	Percentage
**Sex**	**N=6679**	195 (2.8)		
Male			2792	41.8
Female			3887	58.2
**Admitting ward**	**N=5310**	1564 (22.8)		
Medical			3643	68.6
Emergency medicine department/ICU			19	0.4
Gynecological wards			290	5.5
Pediatric wards			531	10.0
Psychiatry wards			11	0.2
Surgical wards			795	15.0
Obstetric wards			21	0.4
**Age groups**	**N=6732**	142(2.1)		
0-4			496	7.4
5-17			340	5.0
18-25			515	7.7
26-35			1968	29.2
36-45			1926	28.6
46-55			937	13.9
56+			550	8.2
**ART use**	**N=6704**	170 (2.5)*		
Yes			4132	61.6
No			2572	38.4

ICU=Intensive Care Unit

* ART status unknown

Out of 6874 reviewed patient's files, only 6482(94.3%) had documented discharge diagnoses. The leading diagnoses were tuberculosis 1396/6482 (21.5%), followed by anaemia 1016/6482 (15.6%), malignancies 789/6482 (12.2%), central nervous system (CNS) infections 541/6482 (8.3%), chronic kidney disease 308/6482 (4.8%), while cardiovascular diseases and diarrhoea each accounted 3.6% (235/6482 and 231/6482 respectively). Chest infections other than tuberculosis were seen in 218/6482 (3.4%) while other forms of infections were seen in 206/6482 (3.2%) of the total admissions. (See Table 3)

**TABLE 2.** Causes of Admissions of HIV Infected Patients in Muhimbili National Hospital From June 2005 to June 2015, N= 6482*

SN.	Diagnosis	Frequency	Percentage
1.	Tuberculosis of any form	1396	21.5
2.	Anemias	1016	15.6
3.	Cancer	789	12.2
4.	CNS infections	541	8.3
5.	Chronic kidney disease (CKD)	308	4.8
6.	Cardiovascular diseases	235	3.6
7.	Chronic Diarrhea	231	3.6
8.	Chest infections other than Tuberculosis	218	3.4
9.	Other Infections	206	3.2
10.	Malnutrition	198	3.0
11.	Surgical conditions other than cancer	161	2.5
12.	Noninfectious Central nervous system diseases	159	2.5
13.	Encephalopathy from any cause	155	2.4
14.	Oral/Esophageal candidiasis	118	1.8
15.	Psychiatric disorders	82	1.3
16.	Peptic Ulcer Disease	73	1.1
17.	Unspecified diagnosis in HIV	64	1.0
18.	Skin diseases (noninfectious)	64	1.0
19.	Liver diseases (noninfectious)	49	0.8
20.	Septicemia	47	0.7
21.	Others	372	5.7

CKD included diagnoses such as CKD, Nephrotic syndrome and HIV associated nephropathy (HIVAN)

Liver diseases included all liver diseases other than viral hepatitis and malignancies

Genital viral infections included genital herpes and genital warts

ENT= Ear, Nose and throat

* Only 6482 patients had documented discharge diagnoses

## DISCUSSION

The trend of HIV admissions at Muhimbili National Hospital showed a drastic drop from year 2005 to 2009 then plateaued to year 2015. The drop in number of patient admissions can be largely attributable to ART use and a decrease in HIV prevalence in the general population. However, the drop might as well be due to the fact that other public and private facilities are now more willing to hospitalize people with HIV disease. The present analysis has shown that there had been a surge of admissions among HIV negative individuals between years 2007 and 2014 probably due to more admissions of patients with non-communicable diseases owing to full operationalised cardiac building and renal services in the hospital which were not there before. Furthermore, the hospital bed capacity has expanded owing to existence of the new Paediatric block and the release of 2 wards from Muhimbili Orthopaedic Institute to Muhimbili National hospital. This surge can also partly be explained by the growth of the city's population which has almost doubled from the 2,487,288 people in 2002 to 4,364,541 population in 2012. It is estimated that there were 5,166,570 million people in Dar es Salaam in 2015.^25,26^

We have had an increase in Non-Communicable Diseases (NCDs) like diabetes mellitus, hypertension and chronic kidney disease due to an increase in aging population. Generally, national HIV surveys had shown a decrease in HIV prevalence in Tanzania from 7.2% in 2004 ^20^ to 5.1% in 2012.^21^ This drop of prevalence has been reported in neighbouring countries as well as in most of Sub-Saharan countries.^2,27,28^ In Kenya HIV prevalence dropped from 7.2% in 2007 to 5.6% in 2012^28^ whilst in Uganda HIV prevalence dropped from 7.8% at the first survey round 1989/1990 ^29^ to 6.2% in 2017.^27^ All these findings are consistent with the findings of the present study.

Admissions from female patients were predominant, so were medical admissions and adult admissions. Predominance of female and adult admissions is attributable to higher rates of HIV infection among females than males and among adults than children in Tanzania.^21^ The median CD4 count was low indicating that HIV hospital admissions were predominantly among those with severe immunosuppression. The causes of admissions among HIV infected patients were largely tuberculosis, anaemia and cancers in that order, collectively constituting 49.3% of all admissions. Since the advent of HIV infection tuberculosis has remained the number one cause of morbidity and mortality among HIV infected patients.^30,31^ Tuberculosis has also remained the major cause of admission in Kenya.

In the present study, diarrhoea ranked the 7^th^ as the cause of admissions. This is a change of pattern from the situation in the pre-ART era when chronic diarrhoea used to be one of the most common complaints among HIV infected patients. Opportunistic infections' (OIs) pattern in the pre-ART era in Uganda showed that diarrhoea<1 month (30.6%) was the second common OI after oral candidiasis (34.6%) followed by geohelminths (26.5%), *M.tuberculosis* (17.7%), malaria (15.1%) and bacterial pneumonia (11.2%). In the Ugandan case, ART was able to change the pattern to geohelminths (32.4%), diarrhoea<1 month (25.6%), *M.tuberculosis* (18.2%) and oral candida (18.1%).^33^

Among cancer admissions, Kaposi's sarcoma (KS) was the most common cause of admissions. Kaposi's sarcoma has been reported to have declined in other parts of the world. In Europe and U.S.A the rates of KS have declined by 30% to 50% since the introduction of ART.^34^ Despite Tanzania's achievement of the 2015 millennium goal number 6c of reducing 50% of TB prevalence and deaths from the 1990 values in the year 2013,^35^ tuberculosis remained the leading cause of admissions among HIV infected patients. In the present study TB was the number one cause of admission. It has been found that the rate of HIV-TB co-infection depends on the rate of HIV infection in the community.^36^ This fact explains why TB is still rampant, even after a drop of HIV prevalence in Tanzania. TB was the most common opportunistic infection in a study in India^37^ and the third common opportunistic infection in Ethiopia.^38^

Anaemia was the second most common cause of admission, having been mainly secondary to HIV infection itself, HIV drug-induced or treatment failure. A study in Ethiopia found that Zidovudine-based ART and duration on ART predicted anaemia among ART experienced patients while presence of opportunistic infections (tuberculosis being among them) and rural residence predicted presence of anaemia among ART naïve patients.^39^ Anaemia has been found to be an independent risk factor for death among HIV-infected patients^40^ and among HIV/TB co-infected patients.^41^

### Limitations

Due to inadequate documentation, some information from the hard copy files were missing. Files of some patients were nowhere to be seen due to inadequate filing system and record keeping of the paper based files. Since HIV testing was not done to all admitted patients, it is possible that some patients might had been hospitalized with HIV related diseases but were categorised as HIV uninfected, and thus leading to under estimation of proportion of HIV related admissions.

## CONCLUSION

There has been a drastic drop of HIV related admissions at Muhimbili National Hospital from the year 2005 despite the fact that total hospital admissions have been on increase. HIV remains a problem of the adults, largely females. HIV infected patients were more likely to be admitted with medical conditions than others. Severe immunosuppression was prominent among the admissions. Despite the availability of ART and effective anti-tubercular drugs, tuberculosis remained the most common opportunistic infection and a cause of admission. Anaemia and cancers have become more important causes of admission than were diarrhoea and other infections, which had been the most common conditions in the preART era.
